# Intratumoral Microbiota Changes with Tumor Stage and Influences the Immune Signature of Oral Squamous Cell Carcinoma

**DOI:** 10.1128/spectrum.04596-22

**Published:** 2023-07-06

**Authors:** Raghwendra Pratap Singh, Naina Kumari, Sameer Gupta, Riddhi Jaiswal, Divya Mehrotra, Sudhir Singh, Souvik Mukherjee, Rashmi Kumar

**Affiliations:** a Immunology Laboratory, Council for Scientific and Industrial Research-Institute of Microbial Technology, Chandigarh, India; b Academy of Scientific and Innovative Research (AcSIR), Ghaziabad, India; c Human Microbiome Research Laboratory, National Institute of Biomedical Genomics, Kalyani, West-Bengal, India; d Department of Surgical Oncology, King George’s Medical University, Lucknow, Uttar Pradesh, India; e Department of Pathology, King George’s Medical University, Lucknow, Uttar Pradesh, India; f Department of Oral and Maxillofacial Surgery, Faculty of Dental Sciences, King George’s Medical University, Lucknow, Uttar Pradesh, India; g Department of Radiology, King George’s Medical University, Lucknow, Uttar Pradesh, India; Nanchang University

**Keywords:** microbiome, tumor microenvironment, inflammation, OSCC, biomarker

## Abstract

Characterization of the oral microbiota profile through various studies has shown an association between the microbiome and oral cancer; however, stage-specific determinants of dynamic changes in microbial communities of oral cancer remain elusive. Additionally, the influence of the intratumoral microbiota on the intratumoral immune system remains largely unexplored. Therefore, this study aims to stratify microbial abundance in the early-onset and subsequent stages of oral cancer and analyze their influence on clinical-pathological and immunological features. The microbiome composition of tissue biopsy samples was identified using 16S rRNA amplicon sequencing, while intratumoral and systemic immune profiling was done with flow cytometry and immunohistochemistry-based analysis. The bacterial composition differed significantly among precancer, early cancer, and late cancer stages with the enrichment of genera *Capnocytophaga*, *Fusobacterium*, and Treponema in the cancer group, while Streptococcus and *Rothia* were enriched in the precancer group. Late cancer stages were significantly associated with *Capnocytophaga* with high predicting accuracy, while *Fusobacterium* was associated with early stages of cancer. A dense intermicrobial and microbiome-immune network was observed in the precancer group. At the cellular level, intratumoral immune cell infiltration of B cells and T cells (CD4^+^ and CD8^+^) was observed with enrichment of the effector memory phenotype. Naive and effector subsets of tumor-infiltrating lymphocytes (TILs) and related gene expression were found to be distinctly associated with bacterial communities; most importantly, highly abundant bacterial genera of the tumor microenvironment were either negatively correlated or not associated with the effector lymphocytes, which led to the conclusion that the tumor microenvironment favors an immunosuppressive and nonimmunogenic microbiota.

**IMPORTANCE** The gut microbiome has been explored extensively for its importance in the modulation of systemic inflammation and immune response; in contrast, the intratumoral microbiome is less studied for its influence on immunity in cancer. Given the established correlation between intratumoral lymphocyte infiltration and patient survival in cases of solid tumors, it was pertinent to explore the extrinsic factor influencing immune cell infiltration in the tumor. Modulation of intratumoral microbiota could have a beneficial effect on the antitumor immune response. This study stratifies the microbial profile of oral squamous cell carcinoma starting from precancer to late-stage cancer and provides evidence for their immunomodulatory role in the tumor microenvironment. Our results suggest combining microbiome study with immunological signatures of tumors for their prognostic and diagnostic application.

## INTRODUCTION

Lip and oral cavity cancer is the 17th most prevalent cancer worldwide and the 2nd most prevalent cancer in India, representing 10.3% of total cancer incidences. It is the most prevalent cancer in males and the 5th most prevalent cancer among females (1, 2). Among all types of head and neck cancer, oral squamous cell carcinoma (OSCC) is the most common form amounting to 80% to 90% of all oral cavity neoplasms (3, 4). Despite the advances in multimodal treatment options available for oral cancer, including surgical resection, adjuvant chemotherapy, and radiotherapy, it has a poor prognosis rate with a 5-year overall survival of 60%, owing to late diagnosis mostly after lymph node metastasis (5, 6). Prominent risk factors for OSCC include smoking, alcohol consumption, and human papillomavirus (HPV) infection (7, 8); additionally, chewing betel quid and areca nut are significant risk factors in developing countries, including India (9–11). However, the occurrence of oral cancer in nonsmokers and nondrinkers (12) indicates the involvement of other possible factors in influencing the risk of cancer development. Poor oral hygiene, dentition, and chronic infection-related inflammation are factors reported to be associated with the pathogenesis of cancer (13–15). Inflammation in the oral cavity is linked with alteration of the diverse resident microbiome and manifests not only in the context of oral cavity-related ailment-like dental caries and periodontitis but also with diseases of distant organs, such as cardiovascular diseases, gastrointestinal tract, and vaginal infection (16–18). Recently, the connection between microbial dysbiosis and cancer has become the focus of multiple studies, and their association is reported for various cancer types, such as breast, cervical, renal, and colorectal cancer (19–22). The microbiome can regulate tumorigenesis at both local and distant sites by facilitating the metabolism of environmental factors to produce their carcinogenic effect, thereby influencing inflammation and immunity; however, the mechanism is largely elusive (23).

Several studies have characterized the structure and function of the oral microbial community in health and disease (16, 24, 25), and microbial dysbiosis is increasingly considered one of the factors associated with oral cancer. Initial studies utilizing culture-dependent techniques and low-throughput methods have established the association between the bacterial profile and OSCC. The differential presence of *Porphyromonas* spp., *Fusobacterium* spp., and Streptococcus anginosus (26, 27) was reported to be associated with OSCC. Later with pyrosequencing and next-generation sequencing (NGS), a high-throughput sequencing method that enabled simultaneous profiling of a large number of samples for their bacterial composition in significant depth in a culture-free and affordable manner, bacterial association with OSCC was confirmed (28, 29). By using 454 parallel sequencing of the 16S rRNA gene, Hooper et al. (30) have characterized 10 patient tissue samples with phyla-specific primers and identified the enrichment of saccharolytic and aciduric species in cancerous tissue compared with adjacent normal mucosa. Similarly, Pushalkar et al. (31) have identified the enrichment of Streptococcus spp. and *Gemella* spp., among others, at tumor sites, while Granulicatella adiacens was prevalent in nontumor areas. Schmidt et al. (32) reported the depletion of *Firmicutes* (mostly Streptococcus) and *Actinobacteria* (mostly *Rothia*) relative to anatomically matched contralateral normal tissue samples. Al-Hebshi et al. (33) reported an association of inflammatory bacteria Fusobacterium nucleatum and Pseudomonas aeruginosa with OSCC. In a large-scale study, Bornigen et al. (34) studied the effect of multiple factors on the microbial composition and functional profile associated with OSCC and identified tooth loss as one of the crucial factors for cancer initiation along with established risk factors. They reported a differential abundance of certain taxa, especially the enrichment of *Dialister* and depletion of *Scardovia.* Several groups have studied the salivary microbiome to identify noninvasive, potential prognostic biomarkers as an alternate strategy (35, 36). Lee et al. (37) reported a differential abundance of five genera, namely, *Bacillus*, *Enterococcus*, *Parvimonas*, *Peptostreptococcus*, and *Slackia*, between epithelial precursor lesions and cancer, while, Guerrero-Preston et al. (28) reported a relative abundance of Streptococcus, *Dialister*, and *Veillonella* and depletion of *Neisseria*, *Aggregatibacter*, Haemophilus (*Firmicutes*), and *Leptotrichia* (*Fusobacteria*) in tumor samples compared with control samples. All these studies have established that distinct microbial taxa are associated with OSCC; however, oral cancer stage-specific identification of bacterial taxa having prognostic potential is still lacking. This gap in knowledge is primarily due to the unavailability of samples from all stages of cancer in a single study. Moreover, large cohort studies emphasizing functional correlation are warranted for establishing microbial signatures with OSCC development.

Tumor-infiltrating lymphocytes (TILs), including T cells, B cells, and innate immune components, such as macrophages and neutrophils, have been studied and correlated with the prognosis of cancer in the case of solid tumors (38, 39). Regulatory T-cell (Tregs) subsets have been studied extensively and linked with poor prognosis (40). Recently, studies have explored the role of gut microbiota in the modulation of tumor immunity and immunotherapy both at local sites, such as esophageal, gastric, and colorectal carcinoma (41, 42), and at distant sites, such as pancreatic cancer (43). So far, less emphasis has been given to the intratumoral microbiota on extraintestinal tumor development. Few reports are available describing the influence of local microbiota on inflammatory and metabolic responses in the tumor microenvironment in the case of breast and ovarian cancer (19, 44); however, none of the studies have explored the impact of intratumoral microbiota on TILs in the case of oral cancer.

In the current study, we explored the microbial difference among different stages of OSCC. We have included patients from precancer to late-stage cancer and aimed to identify stage-specific microbial signatures. Furthermore, we explored the role of local microbiota-immune system interplay in oral tumorigenesis by comparing the effect of local microbiota on tumor-infiltrating immune cells and cytokine gene expression. Our findings address our hypothesis that the intratumoral microbiota influences the regional immune system in oral cancer, and there is a possibility of immune modulation through microbial manipulation.

## RESULTS

### Study participant characteristics.

This study included a total of 75 patients for microbiome analysis. Intratumoral bacterial diversity profiles were generated from 15 precancer and 60 oral cancer patients, which were further subdivided into early-stage (T1 and T2) and late-stage (T3 and T4) cancer groups, and 20 adjacent tumor tissue (AT) specimens; a total of 95 samples were analyzed. The clinical characteristics of the study subjects are listed in Table 1. No difference in age, gender, smoking status, and alcohol consumption was observed between the groups. Characteristics of taxonomic distribution are presented in detail in Table S1 in the supplemental material.

**TABLE 1:** Patient characteristics and distribution of samples across analysis^*a*^

Parameter	Patient data	Data by test			
		16S rRNA gene sequencing	Flow cytometry analysis	Gene expression analysis	IHC analysis
Sex					
Male	71	58	50	37	43
Female	24	17	13	13	19
Total samples	95	75	63	50	62
Age (mean ± SD)	48 ± 12	50 ± 11	51 ± 9	52 ± 10	51 ± 13
Group of samples					
Cancer	69	60	40 (PBMCs), 22 (TILs)	40	48
Precancer	15	15	12	10	14
Healthy control	11		11		
Localization					
Buccal mucosa	38	33	24	20	21
Tongue	13	11	10	9	8
Upper and lower alveolus	18	16	6	11	19
T stage					
I	15	15	9	10	11
II	15	15	9	10	15
III	15	15	6	10	11
IV	15	15	7	10	11
NA	09		9		
N stage					
Node positive	25	25	12	12	31
Node negative	35	35	19	28	17
NA	09				

a Values are presented as number of patients unless otherwise indicated.

### Microbial diversity changes with disease progression.

**FIG 1:**
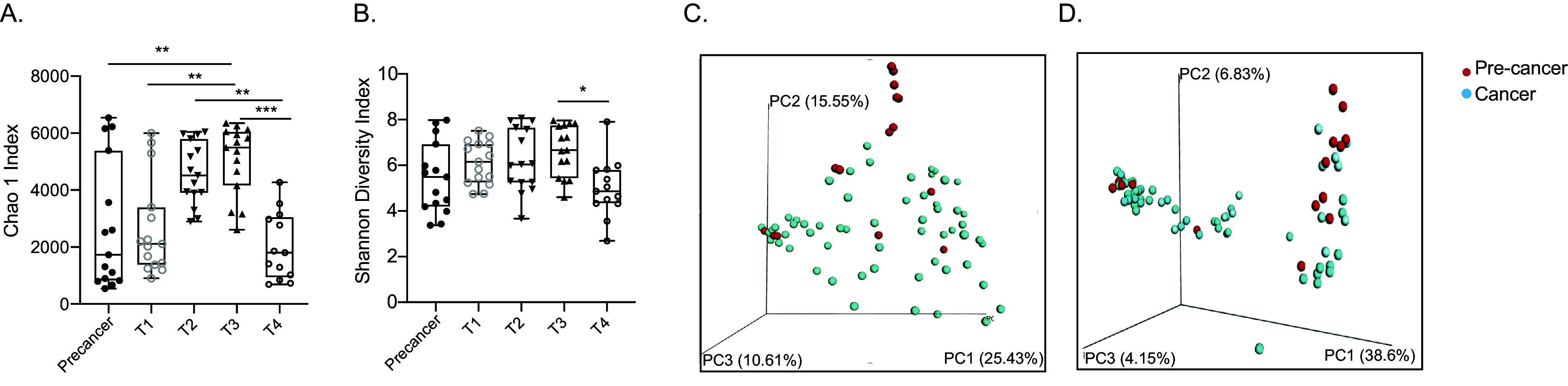
Oral cancer bacterial communities differ with the clinical stage of cancer. Bacterial alpha diversity indices among different stages of oral cancer were measured by Chao1 index (A) and Shannon diversity index (B). Bacterial beta diversity indices between precancer and cancer groups were measured by weighted UniFrac distance (C) and unweighted UniFrac distance (D). Statistical analysis was done by one way ANOVA followed by the Kruskal-Wallis test. *, *P* < 0.05; **, *P* < 0.01; ***, *P* < 0.001.

We analyzed and compared the oral microbiota profiles of precancer and cancer groups further subdivided into the early and late stages of cancer to quantify overall differences in microbial composition. Microbial alpha diversity was determined using richness (Chao 1 index) and diversity (Shannon index) (45) metrics within the groups. The Chao index was significantly higher at the T3 stage compared with that at the precancer (*P* < 0.01) and both T1 (*P* < 0.01) and T4 (*P* < 0.001) stages, while it is similar to the T2 stage (Fig. 1A). The Shannon index was comparable between the groups; however, the T4 stage demonstrated a lesser diversity compared with the T3 stage (*P* < 0.05) (Fig. 1B). Alpha diversity indices were comparable between adjacent tumor tissue (AT) and their tumor counterpart (TT) (see Fig. S1B and C in the supplemental material). These observations suggest a loss of both richness and diversity of tumor-residing bacteria at the T4 stage of cancer. Next, we measured the beta diversity (between samples) to detect the phylogenetic relationship between bacterial communities among different groups to estimate the overall structural features of tumoral microbiota. A weighted UniFrac principal coordinate analysis (PCoA) using Bray-Curtis metric distances (46) was calculated based on the relative abundance of operational taxonomic units (OTUs) (at a 97% similarity), indicating a separated clustering between OTUs from the precancer and cancer cohort (Fig. 1C). The unweighted UniFrac distance measurement for the beta diversity produced similar results (Fig. 1D), suggesting a notable difference in the tumor microbiome at precancer and cancer stages. However, both measures did not reveal any significant difference among adjacent control and tumor samples (see Fig. S1D and E in the supplemental material), suggesting phylogenetic contiguity of tumor microbial communities within each group.

### Taxonomic comparison of tumor microbial communities revealed distinct bacterial composition among different stages of cancer.

**FIG 2:**
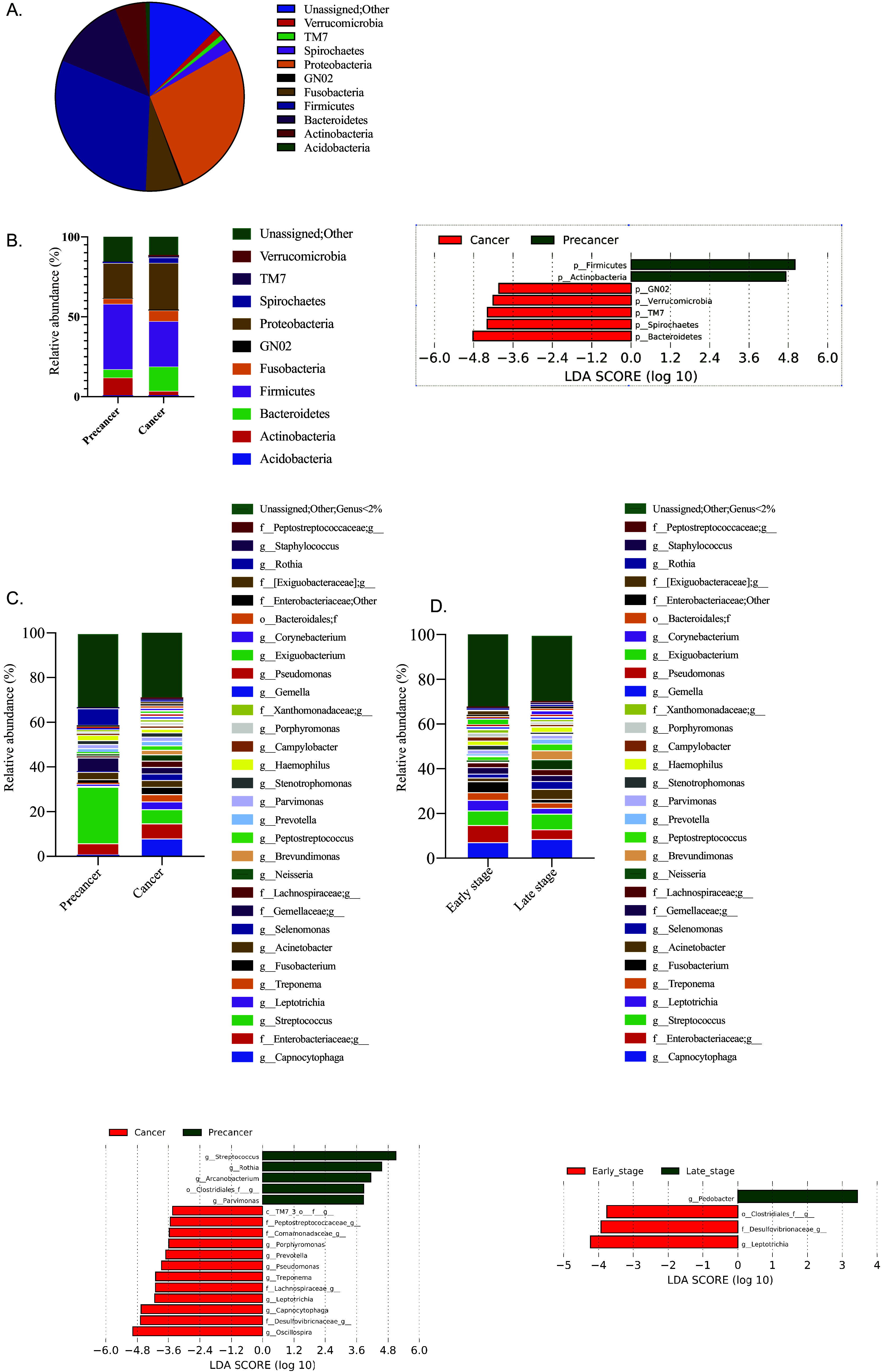
Taxonomic composition of microbiota in OSCC. (A) Relative abundances of core bacterial phyla in the present data set. (B) Relative and differential abundances of core bacterial phyla among precancer and cancer groups. (C) Relative and differential abundances of core bacterial genera among precancer and cancer groups. (D) Relative and differential abundances of core bacterial genera among early cancer and late cancer stages. Differential abundance analysis was done by LEfSe and represented through LDA plot.

We next sought to determine the overall difference in bacterial communities of the tumor microbiome at progressive stages of the disease to find stage-specific biomarkers of OSCC. General intratumoral microbiota composition in all patients revealed the presence of diversified communities (Fig. 2A, combined all samples at the phylum level). *Firmicutes* was the most abundant phylum, followed by *Proteobacteria* and *Bacteroidetes*, constituting almost 70% (all three together) of all sequences. Next, the differential enrichment of specific bacterial communities at various taxonomic levels was compared, focusing on core taxa. Marked differences in the relative abundance of the bacterial communities were observed between the precancer and cancer groups. The three most abundant phyla in precancer samples comprised *Firmicutes* (40.90%), *Proteobacteria* (22.09%), and *Actinobacteria* (11.07%), whereas in cancer samples, *Proteobacteria* (29.39%) and *Firmicutes* (28.38%) were equally abundant followed by *Bacteroidetes* (15.03%) (Fig. 2B). Among 10 core phyla, seven phyla showed significant differences (*P* < 0.05) between precancer and cancer groups through high-dimensional comparisons using linear discriminant analysis of effect size (LEfSe) analysis (47). The phyla TM7, *GNO2*, *Verrucomicrobia*, *Spirochetes*, and *Bacteroidetes* were significantly increased in cancer groups, while phylae *Actinobacteria* and *Firmicutes* were significantly decreased (Fig. 2B, right). Early- and late-stage cancer had comparable abundances of bacteria; however, a significantly higher abundance of the phylum *Fusobacteria* was associated with early stages of cancer (see Fig. S2A in the supplemental material). We did not observe much difference in tumor tissue (TT) and adjacent tumor tissue (AT) samples; only phylum *Proteobacteria* was significantly increased in the AT samples (see Fig S2B in supplemental material). Compared with precancer samples, *Spirochetes*, *Bacteroidetes*, and *TM7* were enriched in AT samples (see Fig S2C in supplemental material), showing their similarity with cancer samples.

At the family level, 30 families present with more than 2% abundance in any one group of samples were analyzed. In precancer samples, 45% of the total abundance was contributed by eight dominant families, where *Streptococcaceae* was the most abundant family. In cancer samples,18 families had more than 2% abundance, and *Flavobacteriaceae* and *Enterobacteriaceae* were the most abundant. Altogether 13 families showed significant differences (*P* < 0.05) in abundance between the precancer and cancer groups. *Streptococcaceae*, *Micrococcaceae*, and an unidentified family of the *Clostridiales* order were significantly increased in the precancer group, while the remaining 10 families were increased in the cancer group (Fig. S2D). Family *Enterobacteriaceae* was present in equal abundance in both groups, which can be considered common microbiota in the oral cavity.

At the genus level, Streptococcus (10.5%) and *Capnocytophaga* (6.4%) were detected as the most abundant genera (Fig. 2C). Seventeen genera showed significant differences (*P* < 0.05) between precancer and cancer groups through LEfSe analysis. *Capnocytophaga*; *Leptotrichia*; Treponema; *Oscillospira*; Pseudomonas; *Prevotella*; *Porphyromonas*; and an unidentified genera of families *Lachnospiraceae Comamonadaceae*, *Peptostreptococcaceae*, and *Desulfovibrionaceae* showed a significant increase in the cancer groups. In contrast, *Rothia*, Streptococcus, *Arcanobacterium*, *Parvimonas*, and genus of the order *Clostridiales* showed a significant increase in the precancer group (*P* < 0.05) (Fig. 2C). The genus *Pedobacter* was enriched at the early stage, while differential abundances of *Leptotrichia*, a genus of family *Desulfovibrionaceae* and order *Clostridiales*, were associated with late cancer stages (Fig. 2D). LefSe analysis of adjacent tumor (AT) samples with tumor (TT) samples and precancer samples revealed many similarities between AT with tumor, however, AT samples were very different in microbial complexity in comparison to precancer samples (see Fig. S3A and B in the supplemental material).

**FIG 3:**
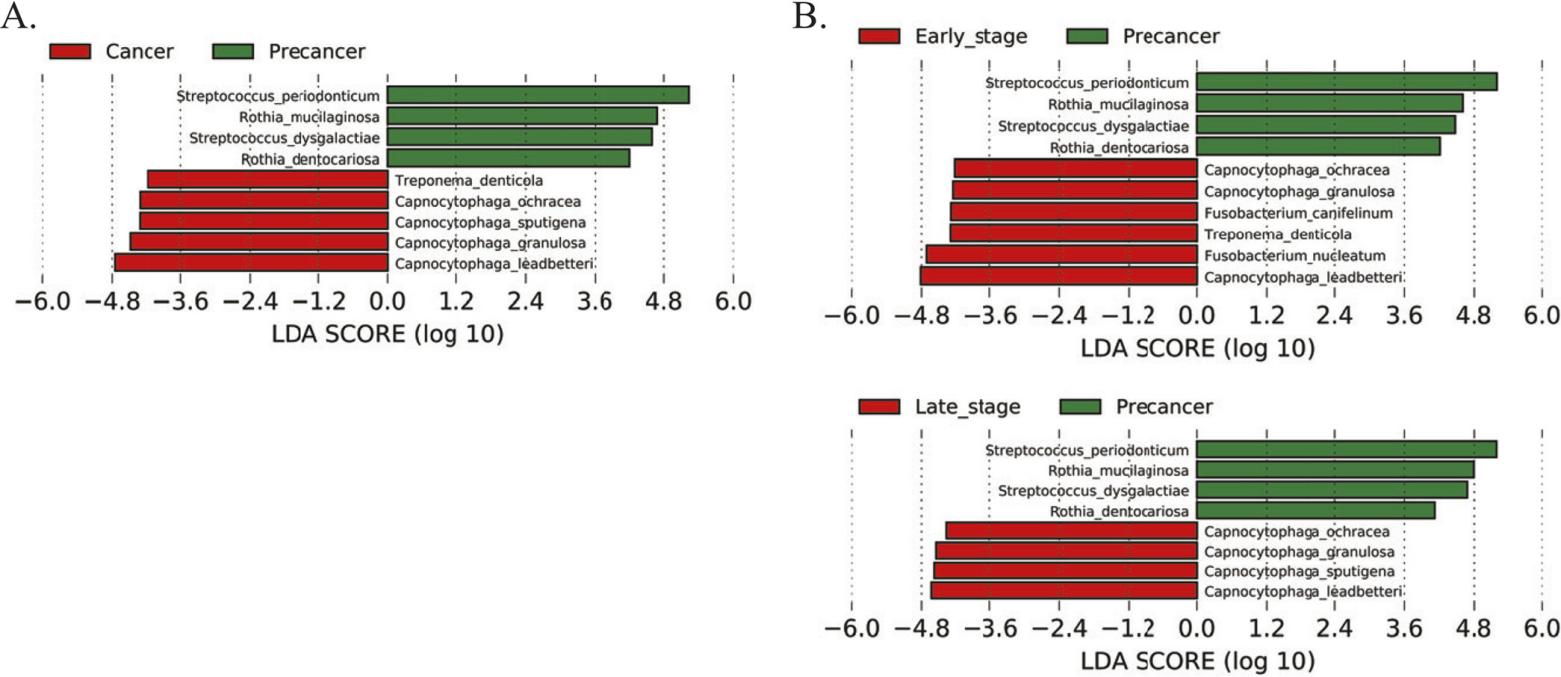
Taxonomic composition of microbiota in OSCC. (A) Differential abundances of core bacterial species among precancer and cancer groups. (B) Differential abundances of core bacterial species between the precancer group with early (T1 and T2) and late cancer (T3 and T4) groups. Differential abundance analysis was done by LEfSe and represented through LDA plot.

Species-level identification was performed with genera with a relative abundance of >5% in any group. A total of 10 such genera were taken into consideration, and 67 species were identified, of which 19 species were assigned as core species having a relative abundance of >1% in any sample. The relative abundance of 15 species was more than 2%; together, they accounted for 85% of the sequences (see Table S2 in the supplemental material). Precancer samples were enriched for the species of Streptococcus and *Rothia*, while species of *Capnocytophaga* were enriched in cancer samples (Fig. 3A). In comparison to precancer samples, early- and late-stage cancer showed a distinct enrichment of bacterial communities. Species of *Fusobacterium*, namely, Fusobacterium canifelinum and F. nucleatum, were enriched at the early stage, while the late-stage of cancer was found to be particularly enriched with species of genus *Capnocytophaga*, namely, Capnocytophaga ochracea, Capnocytophaga granulosa, Capnocytophaga sputigena, and Capnocytophaga leadbatteri (Fig. 3B). However, *C. granulosa* and *C. leadbatteri* were enriched in cancer samples across all stages. *F. canifelinum* was the only differential species in the early and late stages of cancer (Fig. S3E). Next, AT specimens were compared with TT and precancer specimens; enrichment of Rothia mucilaginosa, Rothia dentocariosa, and Neisseria mucosa were detected in AT samples (Fig. S3C) while they differ with precancer samples due to the enrichment of cancer-associated bacterium (Fig. S3D).

### Microbial co-occurrence analysis and prediction of biomarker potential.

**FIG 4:**
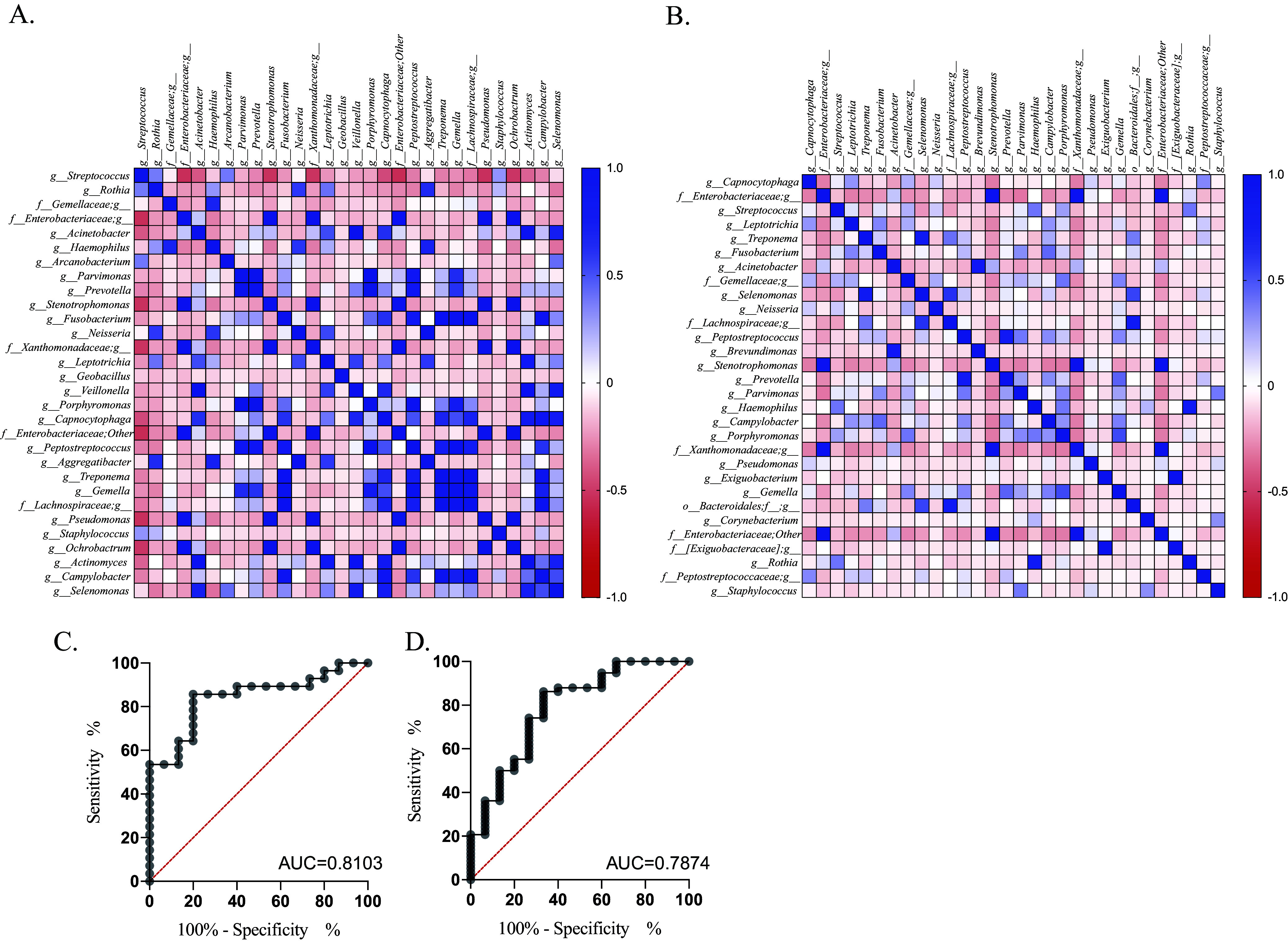
Co-occurrence and prediction analysis. Co-occurrence analysis of top 30 bacterial genera in precancer (A) and cancer (B) groups with Pearson’s correlation analysis presented through heat map. The color key represents *r* values. ROC curve for genus *Capnocytophaga* (C) and Streptococcus (D).

We studied the community relationships among bacteria in the tumoral microenvironment with the 30 most abundant bacterial genera of precancer and cancer samples by calculating Pearson’s correlations among them. They were visualized through a heat map (Fig. 4A and B) and with Cytoscape (v3.9.0) (48) for all correlations with *r* values between +0.5 and −0.5. (see Fig. S4A and B in the supplemental material). Overall, only a few negative correlations were observed in the precancer samples, while all identified bacterial associations were strongly positive in both cases. Altogether, we spotted 27 nodes and 73 edges in precancer samples, while only 18 nodes with 15 edges were observed for cancer samples (Fig. S4 A and B). The most closely associated cluster was formed among *Stenotrophomonas*, Pseudomonas, *Ochrobactrum*, and unidentified genera of *Enterobacteriaceae* and *Xanthomonadaceae* families in the precancer group. In this hub, every bacterial genus is associated with each other with a strong positive correlation (*r* > 0.9). *Stenotrophomonas* and an unidentified genus of family *Xanthomonadaceae* were most positively correlated (*r* = 0.998, *P* < 0.0001). Interestingly, Streptococcus was found to be negatively associated with components of this cluster. *Fusobacterium* formed another hub and was associated with multiple genera, namely, *Capnocytophaga*, *Peptostreptococcus*, Treponema, *Gemella*, Campylobacter, *Bacteroides* and genera of *Comamonadaceae* and *Lachnospiraceae* families; all these associations showed a strong positive correlation (*r* = >0.7 to 0.9). While we observed strong and multiple associations among bacterial genera in precancer samples, these connections were sparse in cancer samples with few strong positive associations. Among these associations, the most positively correlated genera were *Stenotrophomonas* with the genera of families *Xanthomonadaceae* (*r* = 0.99) and *Enterobacteriaceae* (*r* = 0.89) (Fig. 4B) as observed in the precancer group; however, in this case, *Ochrobactrum* and Pseudomonas were not included within the same cluster as observed in the precancer group (Fig. 4A).

Next, to explore the predictive potential of most differential taxa as a diagnostic marker for cancer, we performed receiver operating characteristic (ROC) curve analysis. Taxa were selected based on the LEfSe analysis at the genus level (Fig. 2C), and the five most abundant and differential genera, namely, Streptococcus and *Rothia* of precancer samples and *Capnocytophaga*, *Leptotrichia*, and Treponema of cancer samples, were included for analysis. This analysis gave a predictive accuracy to *Capnocytophaga* (Fig. 4C) and Streptococcus (Fig. 4D) genera for cancer and precancer stage, respectively. The area under the curve (AUC) value (see Table S3 in the supplemental material) reached 0.8103 for *Capnocytophaga* and 0.7874 for Streptococcus, indicating they have good diagnostic potential.

### The intratumoral microbiome influences local immune responses.

**FIG 5:**
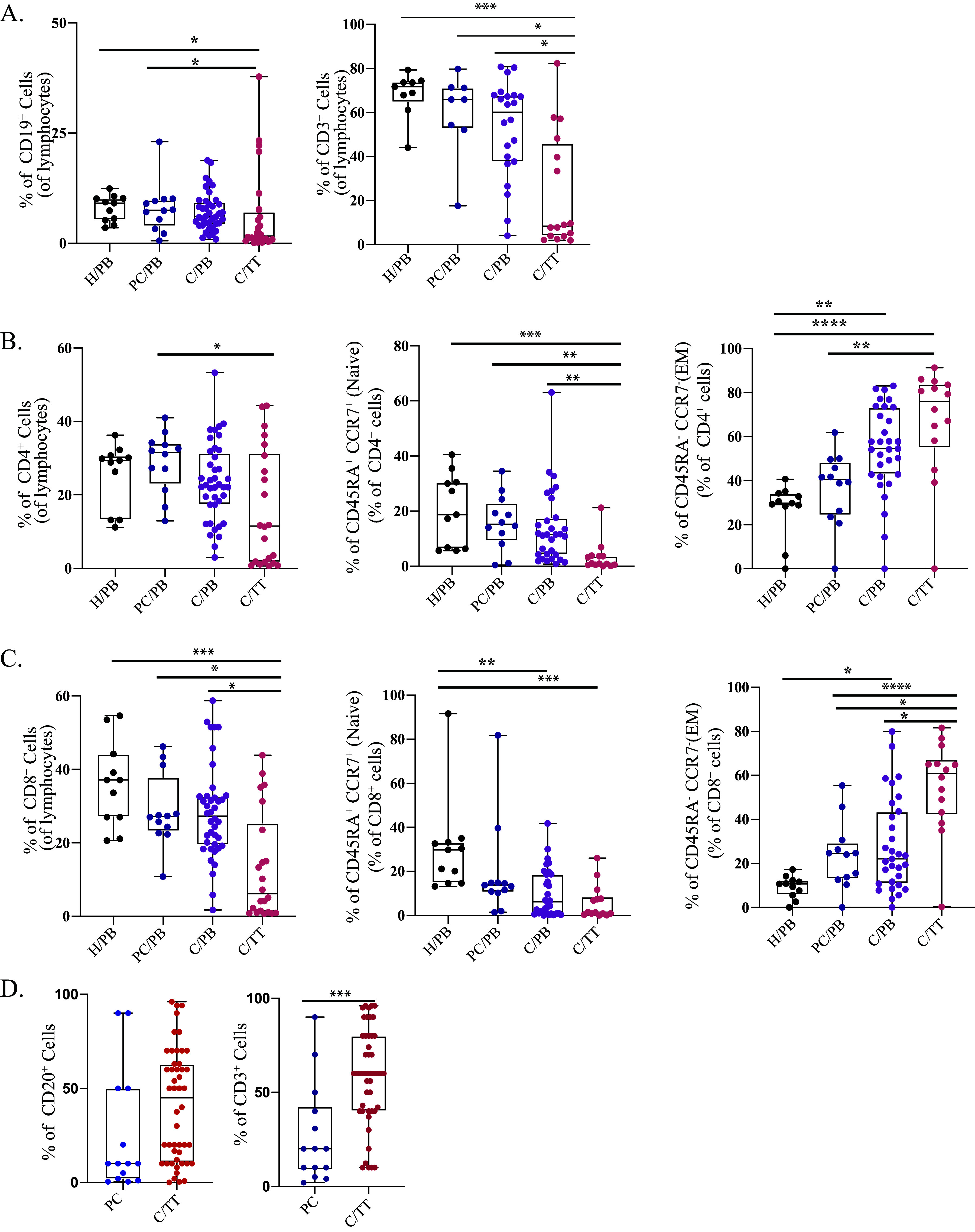
Circulating and tumor-infiltrating lymphocyte composition. (A) Frequency of CD19^+^ B cells and CD3^+^ T cells. (B) Frequency of CD4^+^ T cells, CD4^+^ naive T cells (CD45RA^+^ CCR7^+^), and effector memory (CD45RA^+^ CCR7^−^) subsets. (C) Frequency of CD8^+^ T cells, CD8^+^ naive T cells (CD45RA^+^ CCR7^+^), and effector memory (CD45RA^+^ CCR7^−^) subsets. (D) Frequency (%) of CD20^+^ B cells and CD3^+^ T cells in precancer and cancer groups performed through immunohistochemistry analysis. H/PB, healthy control, peripheral blood; PC/PB, precancer, peripheral blood; C/PB, cancer, peripheral blood; C/TT, cancer, tumor tissue. Statistical analysis was done by one way ANOVA followed by the Kruskal-Wallis test. *, *P* < 0.05; **, *P* < 0.01; ***, *P* < 0.001; ****, *P* < 0.0001.

**FIG 6:**
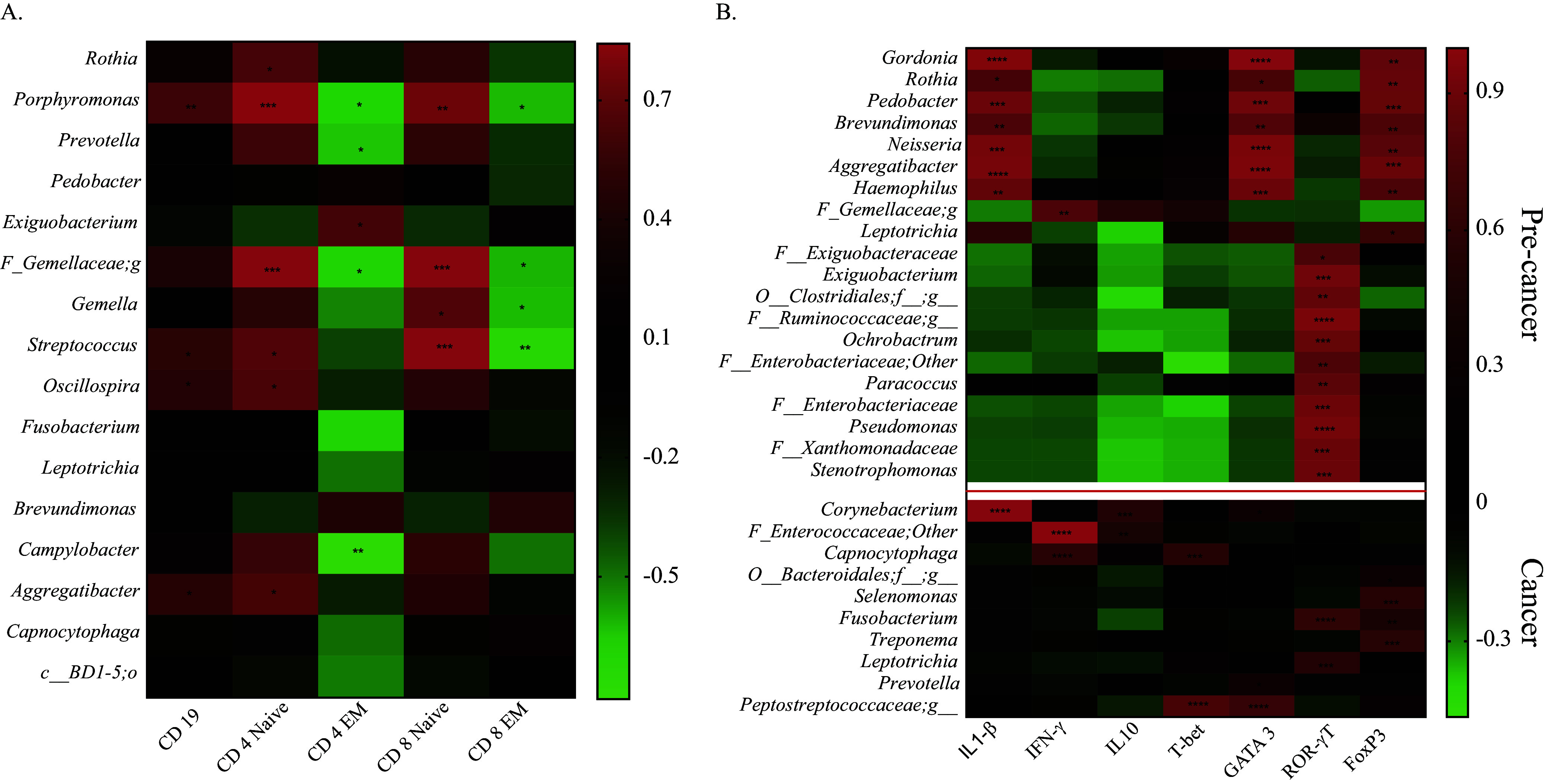
Correlation analysis between microbial abundances and immune markers. (A) Heat map of the Pearson’s correlation analysis of microbial abundances at genus level with tumor-infiltrating lymphocytes. (B) Heat map of the Pearson’s correlation analysis of microbial abundances at genus level with relative expression of immune-related genes. The color key represents *r* values. Significant associations are marked with asterisks; *, *P* < 0.05; **, *P* < 0.01; ***, *P* < 0.001; ****, *P* < 0.0001.

Next, to explore the potential of intratumoral bacterial communities for immunomodulation, we analyzed tumor-infiltrating lymphocytes (TILs) by flow cytometry and immunohistochemistry (IHC). Peripheral blood mononuclear cells from the peripheral blood of all subjects and TILs from tumor biopsy samples from a subset of patients underwent multicolor flow cytometry-based immunophenotyping analysis. Our study observed T and B cell infiltration in the tumor microenvironment (TME) (Fig. 5A). Furthermore, we analyzed the TILs for their T cell subsets and identified naive and memory cells based on CCR7 and CD45RA cell surface markers. Both CD4^+^ and CD8^+^ T cell subsets were enriched for memory phenotype (Fig. 5B and C). The presence of CD3^+^ and CD19^+^ cells in TME was also confirmed with IHC (Fig. 5D). To further investigate the relationship between intratumoral microbial composition with observed immune-phenotype characteristics, we performed a correlation analysis between the dominant microbial relative abundance at the genus level and the infiltrated immune cells (percentage of immune cells in the TME). The significant association with any immune factor is listed in Table S4 in the supplemental material and plotted on a heatmap (CD19^+^ B cells and naive and effector memory (EM) subsets of CD4^+^ and CD8^+^ T cells). We found a significant positive correlation of CD19^+^ B cells with various taxa, such as *Porphyromonas*, Streptococcus, *Oscillospira*, and *Aggregatibacter* (Fig. 6A). In contrast, total CD4^+^ T cells and CD8^+^ T cells were found to correlate with distinct bacterial communities. Furthermore, we performed this analysis with T cell subsets and observed the correlation of tumor microbiota with naive and effector memory T cells. Interestingly, intratumoral bacteria were found to be positively associated with naive cells while they were mostly negatively associated with effector memory T cells having antitumor activity. Genera *Porphyromonas*, *Prevotella*, *Gemella*, *and* Streptococcus were found to be associated with TILs (both CD4^+^ and CD8^+^), while genera *Rothia*, *Oscillospira*, *Aggregatibacter*, *Exiguobacterium*, *Fusobacterium*, and Campylobacter were associated only with CD4^+^ T cells (Fig. 6A).

Given that we had limited samples for cellular-level phenotyping of intratumoral T cell subsets, we delineated the intratumoral T cell subpopulations at the gene level in our samples. We measured the expression of CD4^+^ T cell-associated transcription factors for the T helper cell family (T_H_1-*Tbet*, T_H_2-*GATA3*, and T_H_17-*RORγT*) and T regulatory cells (Tregs) (*FoxP3*). In addition, using reverse transcriptase quantitative PCR (RT-qPCR), we also measured the expression level of 3 cytokine genes (interleukin-10 [IL-10], interferon gamma [IFN-γ], and IL-1-β) in 40 oral cancer tissue samples and 10 precancerous lesion samples that had paired data for microbiome analysis. We did not observe any significant difference in the expression level of T-cell-associated transcription factors among precancer and cancer groups; however, GATA3 (T_H_2 associated) showed an increased expression level, although not significant, in cancer samples. Among cytokine genes, IL-10 expression was higher in cancer samples than that in precancer samples (see Fig. S5 in the supplemental material).

Pearson’s correlation analysis was performed to identify a correlation between the oral cancer microbiome and immune-related gene expression. We observed distinct association patterns with precancer and cancer samples. In cancer samples, *Capnocytophaga* (*r* = 0.55, *P* = 0.003) and an unidentified genus of family *Peptostreptococcaceae* (*r* = 0.75, *P* = 0.001) were found positively associated with *Tbet* expression, while none of the bacteria associated significantly with *Tbet* in precancer samples. *Corynebacterium*, *Prevotella*, *and Peptostreptococcus* were positively associated with *GATA3* expression in cancer, while its expression in precancer samples was associated with distinct and several bacteria. *Fusobacterium* was positively associated with the expression of *RorγT* and *FoxP3* in cancer samples; however, their expression was associated distinctively in precancer samples (Fig. 6B). *Corynebacterium*, *Peptostreptococcaceae*, *Porphyromonas*, and *Fusobacterium* were significantly positively associated with more than one cytokine gene expression in cancer samples and hence can be considered the top influencer of the intratumoral immune response. In precancer samples, IL-1-β, *GATA-3*, and *FoxP3* gene expression covaried with several bacteria belonging to *Gordonia*, *Rothia*, *Pedobacter*, *Brevundimonas*, *Neisseria*, *Aggregatibacter*, and Haemophilus genera. Additionally, in precancer samples, immune factors were associated with a dense microbial network (more than one interconnected species), while in cancer samples, these associations were sparse (Fig. 6B). These results provide insight into the intratumoral microbiome and immune interaction in oral cancer.

## DISCUSSION

This study aimed to characterize and compare the tumor-resident microbiota at progressive stages of OSCC using 16S rRNA gene sequencing and to evaluate their role in intratumoral immunomodulation. We found a marked microbial signature of early-onset and late stages of cancer; moreover, we show distinct immunomodulatory capacities of tumor-resident microbiota. Our results assign potential predictive power to the tumor-resident microbiota and associate their relative abundances with cancer stage. An analysis of tumor-infiltrating immune cells shows both B and T cell infiltration in TME at their terminally differentiated stage with a memory phenotype. In addition, the association between tumor microbial abundance with local immune signature suggests that the tumor microenvironment favors nonimmunogenic and immune-suppressive bacteria. Our results provide evidence that distinct microbial signatures and interbacterial networks are associated with precancer and late stages of OSCC, and together, they influence intratumoral immunity. Hence, this study advocates combining microbiome study with immunological analysis of tumors for their diagnostic and prognostic potential.

Oral pathogenic bacteria connected with early periodontal disease, such as periodontitis, has been associated with incidences of malignant disease (49). We also observed the presence of periodontitis-correlated taxa (50–52), such as *Fusobacterium*, *Neisseria*, *Prevotella*, Treponema, *Parvimonas*, and *Porphyromonas*, among others in our cohort. Out of these taxa, the tumorigenic role of specific bacteria, such as Capnocytophaga gingivalis, Streptococcus mitis, Prevotella melaninogenica, Fusarium nucleatum, Candida albicans, and Porphyromonas gingivalis (53–55), is well determined. However, a consensus regarding a standard microbial signature for the chronology of the disease is still uncertain. Hence, we included patients from precancer, early-stage (T1 and T2), and late-stage (T3 and T4) cancer along with paired adjacent normal tissues from a subset of patients to mark the shift in microbiota with the change of clinical stage of cancer. The study included tumor tissue biopsy specimens in contrast to saliva (37), swabs (51), and oral wash (56, 57) samples to precisely identify the tumor-invading bacteria and to study their interaction with the intratumoral immune system.

The gut microbiome association with antitumoral immunity and therapeutic interventions for various malignancies is now established (58, 59), emphasizing higher microbial diversity with better clinical outcomes (23). We report a loss of bacterial diversity only with the advanced stage (T4) of cancer, which is in line with other studies on oral cancer (31, 32, 60, 61), establishing a loss of bacterial diversity with cancer. Furthermore, a decreased *Firmicutes*-to-*Bacteroidetes* (F/B) ratio is reported to be associated with cancer, such as liver (62), lung (63), and pancreatic cancer (64). The reduced F/B ratio is related to the production of low concentrations of short-chain fatty acids (SCFAs) influencing epithelial barrier function (65) and consequently affecting systemic immunity and local inflammation (66, 67). Accordingly, we observed a decrease in the F/B ratio with an increase in the relative abundance of proteobacteria supporting inflammation in our oral cancer cohort. A cancer stage-specific shift in bacterial composition was observed, where phylum *Fusobacteria*, along with minor phyla *Verrucomicrobia* and *TM7*, were significantly increased at the early stage of cancer, while GNO2 increased during the late stages. At the genus level, Streptococcus was the most abundant genera in the precancer cohort, where they alone had around 25% abundance, followed by *Rothia*. A significant decrease in the abundance of Streptococcus was observed with cancer progression, while the abundance of *Rothia* was lost entirely; this observation was in line with previous reports (32, 33). In our cohort, *Capnocytophaga* was the most abundant genus in cancer samples, followed by Streptococcus and an unidentified genus of *Enterobacteriaceae*. As we have samples distinguished from early and late stages, we could mark the shift in microbial diversity with a change in tumor stage, while the genus of order *Clostridiales*, family *Desulfovibrionaceae*, and *Leptotrichia* were enriched at early stages, *Pedobacter* was significantly improved at late stages (Fig. 2D). Most of the recent studies have reported an increased abundance of genus *Fusobacterium* in not only oral cancer but other cancers as well (68, 69). We report selective enrichment of *Fusobacterium* at early stages of cancer compared with subsequent stages (Fig. S2A); and from AT, suggesting their prime role in cancer initiation. In continuation, at the species level, a series of Streptococcus *and Rothia* species were found to be associated with the precancer group, while species of *Capnocytophaga* and Treponema were the representative of cancer groups. Treponema denticola, Treponema medium, and Fusobacterium nucleatum were found to be associated with early stages of cancer, while multiple species of *Capnocytophaga* (*C. ochracea*, *C. leadbatteri*, and *C. granulosa*) were the representatives of cancer-associated bacteria across the stages. As per our analysis, the relative abundance of *Capnocytophaga* and Streptococcus can be used for early detection and of OSCC.

Various studies have shown a strong correlation between chronic inflammation and the onset of cancer driven by oral bacteria due to poor oral hygiene, which could lead to oral microbiota dysbiosis (13, 70–72). Since these studies have also pointed out a shift in microbiota with cancer, including the present study, we hypothesized that this microbiota shift can directly influence the immune mechanism of the tumor microenvironment with disease progression. The effect of local microbiota on intratumoral immune cells has been shown previously in the case of colorectal, gastric, and breast cancer (73, 74); however, this association in oral cancer has not been reported. Decreased microbial diversity (Fig. 1), enrichment of specific bacterial taxa (Fig. 2), and sparse interactions among bacterial taxa (Fig. 4) observed in oral cancer indicate disrupted microbiome-microbiome and microbiome-immune interaction in cancer, leading to immune dysregulation and cancer progression. To study the influence of intratumoral microbiota on the local immune system, we utilized a multipronged strategy to analyze the immune system components at the cellular and gene level. We investigated humoral and cell-mediated immunity through B and T cells. In agreement with previously reported studies, genera *Porphyromonas*, *Prevotella*, *Gemella*, Streptococcus, and *Oscillospira* were immune influencers, as they were significantly associated with both B and T cells. We observed a positive association of *Rothia*, Streptococcus, *Oscillospira*, and *Aggregatibacter* with naive and central memory (CM) T cells, while their abundance was depleted drastically in the tumor microenvironment, suggesting that the loss of these genera could boost tumor growth by downregulating the antitumor adaptive immune response. Effector memory subsets of T cells were also negatively correlated with *Exiguobacterium*, *Fusobacterium*, and Campylobacter. A highly abundant bacteria of the cancer microenvironment, *Capnocytophaga*, was negatively correlated with an effector memory T cell subset (TEMRA) having antitumor activity (75, 76). Other highly abundant bacteria of the TME, such as Acinetobacter, Treponema, and *Enterobacteriaceae* families, remain unassociated with TILs. These observations suggest that TME selectively enriches nonimmunogenic bacteria or bacteria that can suppress immune activation. We have profiled the expression of T cell subset-specific transcription factors to quantify the presence of corresponding T cell subsets indirectly and correlated them with intratumoral microbiota. The Th2-mediated immune response has been considered protumorigenic (77) through anti-inflammatory cytokine secretion of IL-10 and promotion of pro-tumor B cell action (78). We observed a positive correlation of bacterial taxa, such as *Corynebacterium*, *Prevotella*, and the genus of *Peptostreptococcaceae*, with the expression of GATA3 (T_H_2) and IL-10, thereby suggesting their role as immunosuppressors in the tumor microenvironment. IFN-γ is a potent antitumor cytokine having an established role in tumor elimination through various mechanisms (79) and is secreted by multiple cells of the innate and adaptive compartments (80). A correlation was observed between *Capnocytophaga* and taxa of the *Enterococcaceae* family with IFN-γ. However, both taxa show an increased abundance in the tumor microenvironment compared with precancer lesions. Tregs are another important subset of T cells having a pro-tumorigenic activity (81); in our analysis, the expression of their transcription factor FoxP3 correlated with *Fusobacterium*, Treponema, and *Selenomonas*, which are abundant in the tumor microenvironment, indicating their immunosuppressing potential in TME. *Corynebacterium*, *Peptostreptococcus*, *Porphyromonas*, and *Fusobacterium* showed a maximum interaction with immune features.

Besides the distinction in the abundance of microbiota with cancer stages, we also observed a difference in the association of the oral microbiome with immune features at precancer and cancer stages. While *Fusobacterium*, Treponema, *Leptotrichia*, *Corynebacterium*, *Porphyromonas*, and Streptococcus were the major influencer of TILs and immune genes in cancer cases, *Gordonia*, *Pedobacter*, *Neisseria*, Haemophilus, *Ochrobactrum*, *Paracoccus*, Pseudomonas, and *Stenotrophomonas* were found correlated with immune genes in precancer samples. We observed that not only the bacterial presence but also their interbacterial connections had an influential role in immunomodulation. For example, *Stenotrophomonas*, *Xanthomonadaceae*, and *Ochrobactrum* had equal abundance in both precancer and cancer cases; still, their immunomodulatory role was visible only in the case of the precancer group, where they were forming a network. This intermicrobial network was absent in the case of the cancer group suggesting its prime role in cancer initiation.

The present study gives us a comprehensive account of the microbial shift from the precancer stage to late-stage cancer; however, it has some limitations. Although we have employed precautions in the processing of the samples right from aseptic tumor sample collection protocols to DNA extraction, followed by PCR quality control, we could not rule out the contribution of contamination in our data from the tumor surface and laboratory reagent/kits used in this study. To obtain accurate microbiome data from low microbial biomass samples, such as tumor biopsy specimens, we recommend all future studies to include standard sets of negative controls for the environment and sampling associated contaminants as per the RIDE checklist (82) followed by analysis software for data normalization (83). Another limitation of this study is the unavailability of healthy subjects. As it was ethically not possible to include healthy subjects, we started our analysis with precancer samples; hence, the bacterial signature identified is specific to cancer stages compared with that of precancer samples. As we were interested in identifying the tumor-infiltrated bacteria and their effects on the intratumoral immune compartment, we could not consider incorporating surface swabs from healthy controls. The next limitation of our study is the restricted sample size; moreover, we have even fewer samples for simultaneous intratumoral lymphocyte isolation due to the scarcity of mucosal samples to be sufficient for all three analyses (microbiome analysis through 16S rRNA gene sequencing, cellular immunophenotyping, and gene expression). Finally, we had an unavailability of longitudinal samples. In our cross-sectional sample sets, we tried to match the subjects based on their age, sex, risk factor status, and ethnicity. Still, the oral microenvironment might differ among subjects depending upon their disease management, social status, and genetic makeup. A longitudinal sample set can overcome all these limitations and faithfully capture the transition of tumoral bacterial dynamics from one stage to another.

Still, with all these limitations, to our knowledge, this instance is the first one where a correlation between TILs and the oral bacterial population was studied. In conclusion, the results of the current study reveal that the oral microbiota dynamics change with the progression of oral cancer, which affects intratumoral immune activation. Our findings present convincing data where the intratumoral microenvironment selects bacteria having a suppressive effect on the immune system. Our results provide evidence for the use of upregulated *Capnocytophaga* and downregulated Streptococcus bacteria as late-stage cancer biomarkers.

## MATERIALS AND METHODS

### Ethics statement.

The study was approved by the Institutional Human Ethics Committee of Council for Scientific and Industrial Research (CSIR)-Indian Institute of Toxicology Research (IITR), Lucknow, India (reference no. CSIR-IITR/IHEC/Nov/2016/1); King George’s Medical University (KGMU), Lucknow (reference code 80^th^ ECMIIA/P14); and CSIR-Institute of Microbial Technology (IMTECH), Chandigarh, India (no. IEC Jan 2020#3). Written informed consent was obtained from all participating individuals.

### Patient cohort and sample collection.

Samples were collected from the Department of Oral and Maxillofacial Surgery and Surgical Oncology of King George’s Medical University (KGMU), Lucknow, India, between February 2017 and July 2019 from patients with the clinical diagnosis of precancerous lesions and oral squamous cell carcinoma (OSCC) by KGMU pathology. A total of 95 subjects were recruited for this study, which included 11 healthy control subjects, 15 precancerous subjects, and 69 subjects at different stages of disease pathogenesis (T1 to T4). Peripheral blood was collected from all the subjects. Tissue samples were collected by surgical resection from both the cancerous lesions and adjoining normal areas (adjacent control) from 69 patients. Precancerous lesion samples were collected from patients with leukoplakia, fibrosis, and erythroplakia. None of the subjects were on any antibiotic treatment. The subject-specific information, including gender, age, tumor anatomic location, and tumor, node, metastasis (TNM), is presented in Table 1. Before the surgical removal of the tumor, patients were asked to rinse their mouth with saline water followed by betadine. Sterile surgical instruments were used for tumor resection. Resected tumors were surface sterilized by betadine before sampling. Tumor tissue (TT) specimens were obtained from the inner part of the tumor, while adjacent normal tissue (AT) was collected from the negative margin of the tumor. All tissue samples were collected in RPMI 1640 media and transported to the laboratory within 2 h of sample collection. All laboratory procedures were performed aseptically in biosafety cabinets. One part of the tissue sample was stored at −80°C for sequencing, while another part was processed for immunological studies, subject to sufficient amount of tissue availability. The specimens were processed for sequencing after confirmation of the pathological status by the pathologist.

### DNA extraction.

Total DNA was extracted from 10 to 50 mg of precancerous, cancerous, and adjacent tissue using the DNeasy power soil kit (Qiagen, USA) according to the manufacturer’s protocol. The quality and quantity of DNA were assessed using a NanoDrop 2000 instrument (Thermo Fisher Scientific, USA).

### Amplicon library preparation and 16S rRNA gene sequencing.

Total DNA from 95 samples (15 precancer, 60 cancer, and 20 adjacent control samples) were further processed for amplicon-based sequencing of the V3-V4 hypervariable region of the 16S rRNA gene. A universal barcoded primer set, namely, 341F (CCTACGGGNBGCASCAG) and 758R (GACTACNVGGGTATCTAATCC) (84), was used for the amplification of the V3-V4 hypervariable region of the gene. For each sample, 50 ng DNA was amplified with PCR amplification conditions of 95°C for 30 s and then 35 cycles of (i) 95°C for 10 s, (ii) 56°C for 15 s, and (iii) 68°C for 30 s, followed by a final extension at 68°C for 5 min. Amplified products were checked on a 2% agarose gel, and gel purification was done using the GeneJET gel extraction kit (Thermo Fisher Scientific) to remove nonspecific amplifications. The purified product was quantified using a Qubit 3.0 fluorometer (Life Technology, USA). A NEBNext ultra DNA library preparation kit (New England BioLabs, USA) was used for library preparation from 5 ng of the amplified product. Later, the quantification and quality estimation of the library were done with a 2200 TapeStation instrument (Agilent Technologies, Santa Clara, CA). The prepared library was sequenced with an Illumina HiSeq 2500 instrument (San Diego, CA). Sample preparation and sequencing were performed at Agrigenome Labs Private Limited, Kerala, India.

### Bioinformatics and statistical analysis of microbiome sequences.

Demultiplexed FASTQ files for the paired-end sequences of the 16S rRNA gene of each sample were merged using the Fastq-join module of Quantitative Insights Into Microbial Ecology (QIIME) 1.9.1 (85) after trimming 10 bases from the 3′ ends of both read1 (R1) and read2 (R2) based on their FASTQC reports (http://www.bioinformatics.babraham.ac.uk/projects/fastqc). The merged sequences were subjected to the removal of sequencing primers, and an initial quality control was done based on the following criteria: (i) average read length, ≥400 and ≤1,000 bp; (ii) average quality score, ≥25; (iii) a maximum number of ambiguous base (N), <6; and (iv) a maximum number of forward and reverse primer mismatches of 3. Chimeric sequences were removed from the quality-filtered merged reads, and nonchimeric DNA sequences were clustered into operational taxonomic units (OTUs) with 97% sequence similarity among all the sequences in each OTU using USEARCH (86). The OTUs with only one read were removed before analysis. Taxonomic assignment of the nonchimeric representative sequences from each OTU was done by the UCLUST module of QIIME 1.9.1 based on the Greengenes (v13_8) 16S database (87), and an OTU table (file consisting of reads for each sample along with taxonomic assignments for each OTU) was generated. Community richness, evenness, and diversity (Chao and Shannon indices) (88) were performed using QIIME 1.9.1 and plotted with GraphPad Prism (v8.4.2).

Beta diversity was estimated as Jaccard and Bray-Curtis indices (89) for community membership and community structure, respectively, using QIIME scripts (90). Principal-coordinate analysis (PCoA) was performed for weighted and unweighted Unifrac plots. The core microbiome was defined as those taxa with a relative abundance of >1% in any group. A QIIME-based analysis of 16S rRNA gene data provides unambiguous classification to the genera level. Species-level classification was performed in this study, with only those genera that have a relative abundance of ≥5% in any group, as reported previously (91). The representative OTUs of the selected genera were aligned by the Bayesian lowest common ancestor (BLCA) tool to the 16S microbial database of NCBI (92). Validation was done by aligning the representative sequences to the NCBI 16S microbial database by BLASTn (93); a ≥80% confidence score in BLCA and ≥95% sequence identity in BLAST were set as the criteria for species-level identification (91). A comparative analysis of microbial taxa among different groups was performed by linear discriminant analysis (LDA) method using linear discriminant analysis of effect size (LEfSe) (47).

### Cell isolation from peripheral blood and tumor tissue.

Peripheral blood was obtained from patients before surgery and age-matched healthy control subjects in EDTA vials (BD Pharmingen). Peripheral blood mononuclear cells (PBMCs) were purified using Ficoll-Paque PLUS medium (GE Healthcare Life Sciences) with density gradient centrifugation.

Freshly resected tissue from the primary tumor was transported to the laboratory in RPMI 1640 medium and processed within 2 h. The fresh tumor tissue was cut into small pieces and then manually minced using a scalpel, and single-cell suspension was obtained by mechanical dissociation of tumor tissue. Afterward, the cells were filtered twice through a 70-μm nylon cell strainer (BD). The filtered cell suspension was diluted 1:1 with lymphocyte medium RPMI 1640, layered on Ficoll-Paque PLUS medium (GE Healthcare Biosciences), and centrifuged at 400 × *g* for 25 min to obtain an enriched fraction of tumor infiltrating lymphocytes (TILs).

### Flow cytometry.

At least 1 × 10^6^ cells from cell suspensions (PBMCs and TILs) were incubated with fluorescently labeled primary monoclonal antibodies (see Table S5 in the supplemental material) diluted in fluorescence-activated cell sorter (FACS) buffer (phosphate saline buffer [PBS] with 1% fetal bovine serum and 0.01% NaN_3_) for 30 min at 4°C followed by washing with PBS. Labeled cells were acquired on a BD FACS Canto II flow cytometer (BD Biosciences). The data were analyzed using FlowJo v10 (FlowJo, LLC).

### RT-qPCR analysis.

The mRNA expression levels of T helper cell subset-associated genes, namely, T-bet for T_H_1, GATA-3 for T_H_2, RORγT for T_H_17, FOXP3 for regulatory T cells (Tregs), and three cytokine genes IL-10, IL-1β, and IFN-γ, were determined by RT-qPCR. Total RNA was isolated from tumor tissue, and precancerous lesions stored in RNA later by using TRIzol reagent (Sigma-Aldrich). One microgram of total RNA was reverse transcribed to prepare cDNA using the high-capacity cDNA reverse transcription kit (Applied Biosystems, Foster City, CA). Real-time PCR was performed with SYBR green PCR master mix (Applied Biosystems, Foster City, CA) in a 7500 fast real-time PCR system (Applied Biosystems) using the following program: initial incubation at 50°C for 20 s, then 95°C for 10 min, and followed by 40 cycles of 95°C for 15s and 60°C for 1 min. Respective primers for amplifying concerned genes and the reference gene (GAPDH) are listed in Table S6 in the supplemental material. Relative mRNA expression for every sample was quantified using the delta cycle threshold (*C_T_*) method, normalized to GAPDH mRNA of the same sample as the reference. Thus, the relative gene expression was calculated as follows: relative gene expression = 2^(^*^CT^* ^target gene)−(^*^CT^* ^reference gene)^.

### Immunohistochemistry.

Paraffin-embedded tissue sections (4 μm) were fixed on silane-coated slides, followed by deparaffinization in xylene for 15 min and rehydration in graded alcohol. Antigen retrieval was done by incubating the slides in Tris-EDTA buffer and microwaving at 98°C for 15 min. They were cooled at room temperature (RT) and washed twice with Tris-buffered saline (TBS) buffer. The slides were then incubated with Dako Peroxidase blocking reagent for 5 min and subsequently stained with the following primary antibodies: CD3 (1:200; Dako, Hamburg, Germany) and CD20 (1:200; Dako) for 90 min at room temperature. Slides were washed twice in TBS buffer and incubated with horseradish peroxidase (HRP)-tagged secondary antibody for 30 min. The tissue sections were immersed in 3,3′-diaminobenzidine tetrahydrochloride (Dako) solution and then counterstained with hematoxylin. Finally, sections were dehydrated with graded alcohol, cleared with xylene, and mounted in DPX solution (Sigma).

### Evaluation and scoring.

All slides were first scanned under ×200 magnification (10× eye piece, 20× objective) with a standard light microscope (Olympus CX33) to determine the tumoral boundaries. Cell-rich peritumoral areas were selected and marked, and TILs were counted under ×400 magnification (10× eyepiece, 40× objective). Necrotic and degenerated areas were discarded. The percentage of positive cells was derived by counting the positively stained cells for an immune cell marker out of the total number of cells in the peritumoral areas. Data are represented as the percentage of positive cells.

### Statistical analysis.

An intergroup comparison of intratumoral, systemic immune cell markers and systemic cytokines was performed using GraphPad software 8.4 (La Jolla, CA). Two groups were compared using a Mann-Whitney U nonparametric test, while multiple groups were compared using Kruskal-Wallis analysis of variance (ANOVA) and Student’s *t* test. A Pearson’s correlation analysis was used to plot the correlation between biological markers (FACS-based data as a percentage and relative gene expression data) and the relative abundance of bacteria at the genus level. A *P* value of <0.05 was considered significant, and positive and negative correlation values were indicated. An association with Pearson’s coefficient (*r*) (>0.2) and significant *P* value (*P* < 0.05) were represented with a heat map using GraphPad software 8.4.2.

### Data availability.

The sequencing data and corresponding metadata from this study have been deposited at the GenBank Sequence Read Archive with the accession number PRJNA813034.
