# Supplementary material for: Intratumoral Microbiota Changes with Tumor Stage and Influences the Immune Signature of Oral Squamous Cell Carcinoma
Source: Microbiol Spectr. 2023 Jul 6;11(4):e04596-22. doi: 10.1128/spectrum.04596-22 (PMC10434029; doi:10.1128/spectrum.04596-22)
Supplement: Supplemental file 1 — Supplemental material. Download spectrum.04596-22-s0001.pdf, PDF file, 5.8 MB [file spectrum.04596-22-s0001.pdf]

Supplemental material

Microbiome data analysis characteristics

After quality filtering, 39.6 million sequences were obtained for subsequent analysis from 56 million paired-end raw reads with an average of 0.19-3.5 million reads per sample. The rarefaction plots for the number of observed species, phylogenetic diversity, Shannon and Chao Indices reached a plateau for all the samples rarified for 0.12 million sequences per sample (Supplementary Figure 1a). This plot indicates sufficient sequencing depth to cover the maximum microbiome diversity, richness, and observed species in each sample for further analysis (1, 2). A total of 21852 OTUs at the 97% sequence similarity were obtained, with 13,650 OTUs in pre-cancer 19502 in cancer and 7207 in the adjacent tumor group. The oral microbiome of the study was assigned to a total of 51 phyla and 1055 genera, of which the core microbiome that signifies taxa with the average relative abundance of equal to or more than 1% in any group was identified at each of the hierarchical levels as follows (a) Phyla: 10 out of 51 (b) Genera: 50 out of 1055 (c) Species: 19 out of 67 identified from 10 genera having a relative abundance of more than 5%. The predominant phyla (top 5) belonged to *Firmicutes* (30.5%), *Proteobacteria* (27.3%), *Bacteroidetes* (12.8%) *Fusobacteria* (6.2%) and *Actinobacteria* (5.2%) while predominant genera (top 5) were *Streptococcus* (10.3%), Capnocytophaga (6.4%), unidentified genera of *Enterobacteriaceae* (5.4%), *Neisseria* (3.3%) and *Leptotrichia* (3.2%) in all samples combined (Supplementary table 1).

1. Mukherjee S, Mitra R, Maitra A, Gupta S, Kumaran S, Chakrabortty A, Majumder PP. 2016. Sebum and Hydration Levels in Specific Regions of Human Face Significantly Predict the Nature and Diversity of Facial Skin Microbiome. Sci Rep 6:36062.
2. Willis AD. 2019. Rarefaction, Alpha Diversity, and Statistics. Front Microbiol 10:2407.

Supplemental figures:

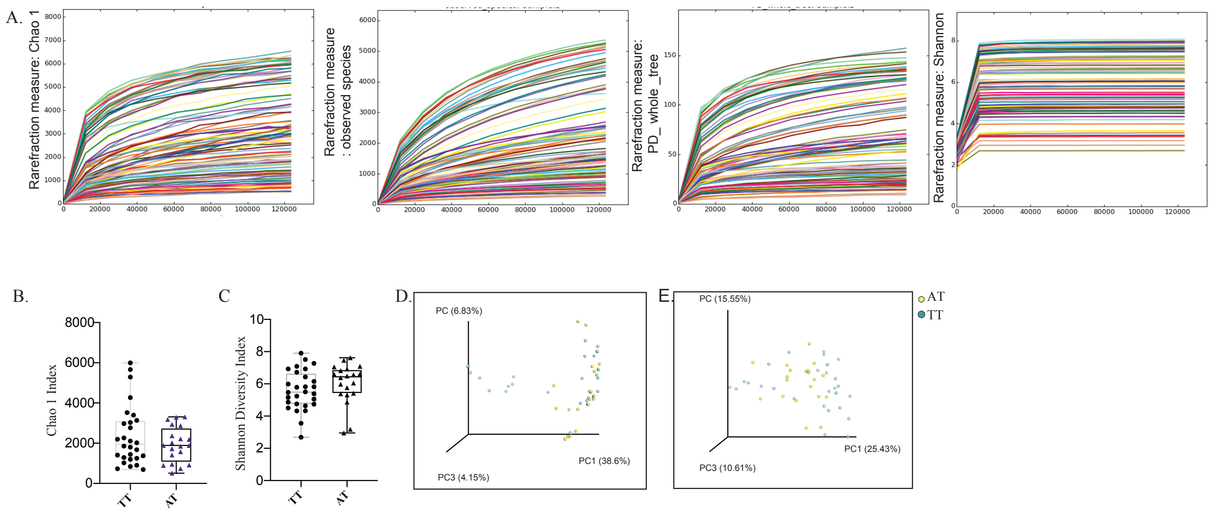

Supplemental figure 1: (A) Rarefaction analysis of 16S rRNA gene sequence obtained in present data set by four measures: Chao1, observed species, PD whole tree and Shannon indices. Bacterial alpha diversity indices among tumor tissue and adjacent tumor tissue samples measured by (B) Chao1 and (C) Shannon diversity indices. Statistical analysis was done by t-test, Mann-Whitney test. Bacterial beta diversity indices between tumor tissue and adjacent tumor tissue samples measured by (D) unweighted UniFrac distance (E) weighted UniFrac distance.

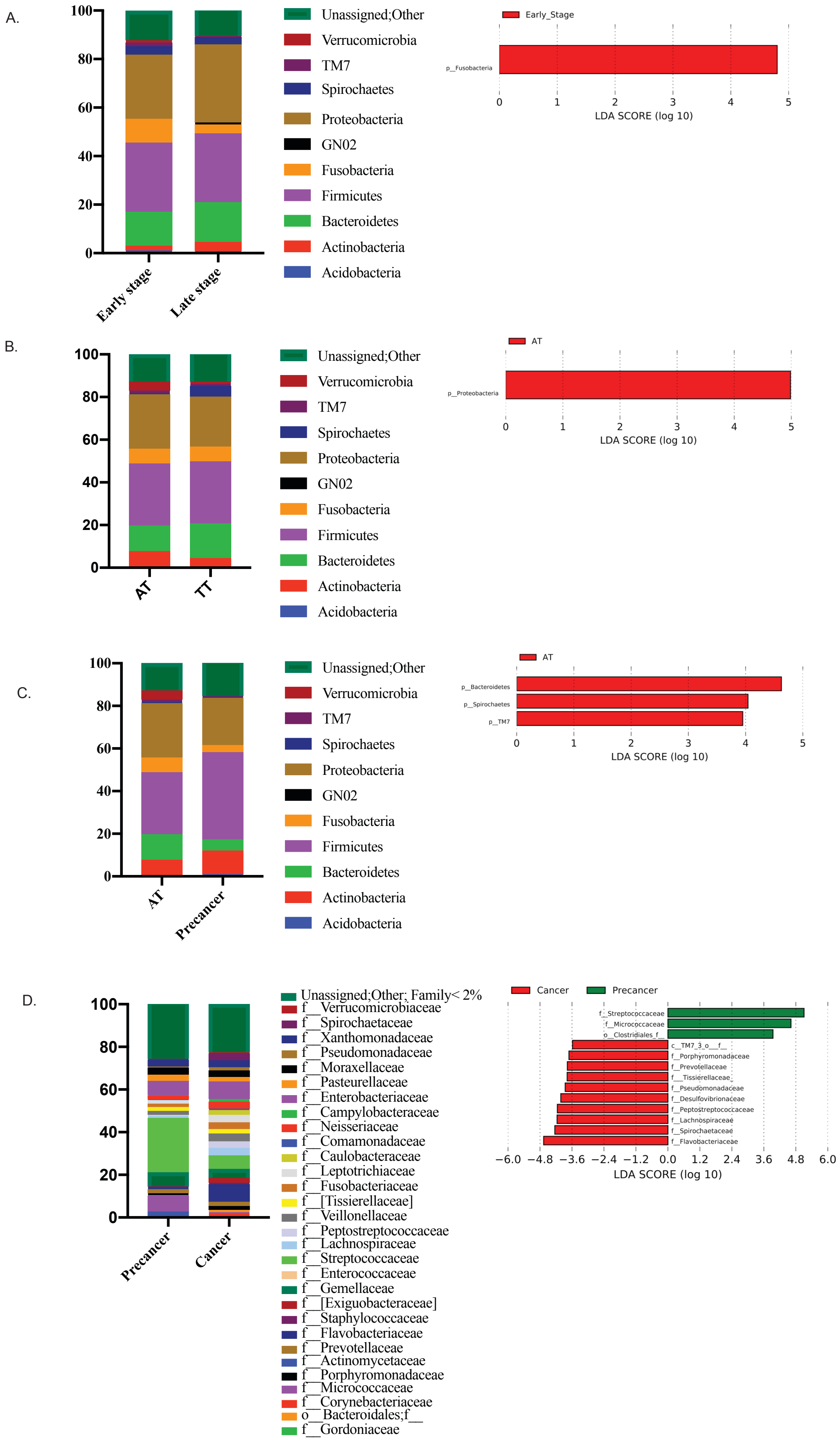
A.
0
20
40
60
80
100
Early stage
Late stage
Unassigned;Other
Verrucomicrobia
TM7
Spirochaetes
Proteobacteria
GN02
Fusobacteria
Firmicutes
Bacteroidetes
Actinobacteria
Acidobacteria
Early_Stage
p__Fusobacteria
0 1 2 3 4 5
LDA SCORE (log 10)
B.
0
20
40
60
80
100
AT
TT
Unassigned;Other
Verrucomicrobia
TM7
Spirochaetes
Proteobacteria
GN02
Fusobacteria
Firmicutes
Bacteroidetes
Actinobacteria
Acidobacteria
AT
p__Proteobacteria
0 1 2 3 4 5
LDA SCORE (log 10)
C.
0
20
40
60
80
100
AT
Precancer
Unassigned;Other
Verrucomicrobia
TM7
Spirochaetes
Proteobacteria
GN02
Fusobacteria
Firmicutes
Bacteroidetes
Actinobacteria
Acidobacteria
AT
p__Bacteroidetes
p__Spirochaetes
p__TM7
0 1 2 3 4 5
LDA SCORE (log 10)
D.
0
20
40
60
80
100
Precancer
Cancer
Unassigned;Other; Family< 2%
f__Verrucomicrobiaceae
f__Spirochaetaceae
f__Xanthomonadaceae
f__Pseudomonadaceae
f__Moraxellaceae
f__Pasteurellaceae
f__Enterobacteriaceae
f__Campylobacteraceae
f__Neisseriaceae
f__Comamonadaceae
f__Caulobacteraceae
f__Leptotrichiaceae
f__Fusobacteriaceae
f__[Tissierellaceae]
f__Veillonellaceae
f__Peptostreptococcaceae
f__Lachnospiraceae
f__Streptococcaceae
f__Enterococcaceae
f__Gemellaceae
f__[Exiguobacteraceae]
f__Staphylococcaceae
f__Flavobacteriaceae
f__Prevotellaceae
f__Actinomycetaceae
f__Porphyromonadaceae
f__Micrococcaceae
f__Corynebacteriaceae
o__Bacteroidales;f__
f__Gordoniaceae
Cancer
Precancer
f__Streptococcaceae
f__Micrococcaceae
o__Clostridiales_f__
c__TM7_3_o___f__
f__Porphyromonadaceae
f__Prevotellaceae
f___Tissierellaceae_
f__Pseudomonadaceae
f__Desulfovibrionaceae
f__Peptostreptococcaceae
f__Lachnospiraceae
f__Spirochaetaceae
f__Flavobacteriaceae
−6.0 −4.8 −3.6 −2.4 −1.2 0.0 1.2 2.4 3.6 4.8 6.0
LDA SCORE (log 10)

Supplemental figure 2: (A) Relative and differential abundances of core bacterial phyla among early (T1 and T2) and late stages (T3 and T4) of cancer groups. (B) Relative and differential abundances of core bacterial phyla among adjacent tumor tissue and tumor tissues. (C) Relative and differential abundances of core bacterial phyla among adjacent tumor tissue and pre-cancer stage. (D) Relative and differential abundances of bacteria at family level among pre-cancer and cancer groups. Differential abundance analysis was done by LEfSe and represented through LDA plot.

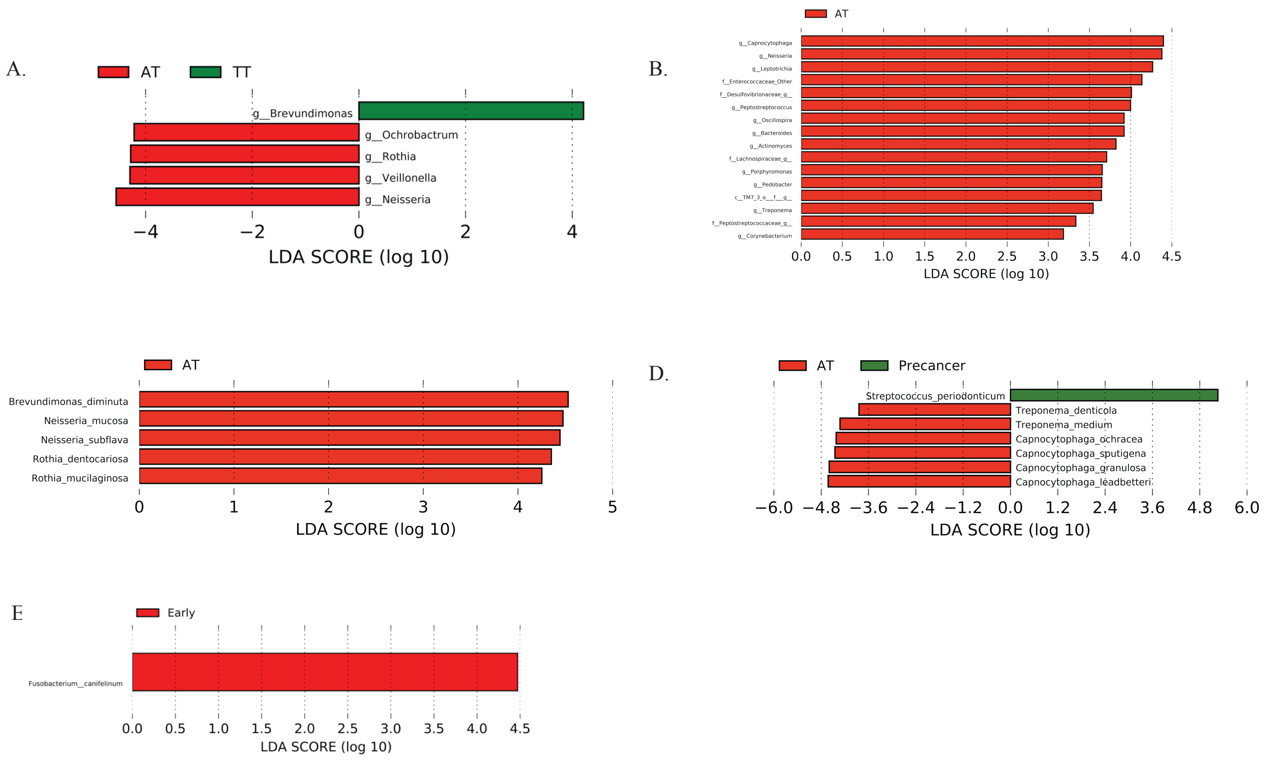

Supplemental figure 3: Differential abundances of core bacterial genera among (A) adjacent tumor tissue (AT) and tumor tissues (TT). (B) pre-cancer and adjacent tumor tissue. Differential abundances of bacteria at species level among adjacent tumor tissue (AT) and (C) tumor tissue (TT) (D) and pre-cancer groups. (E) Differential abundances of bacteria at species level between early (T1and T2) and late cancer stage (T3 and T4) groups. Differential abundance analysis was done by LEfSe and represented through LDA plot.

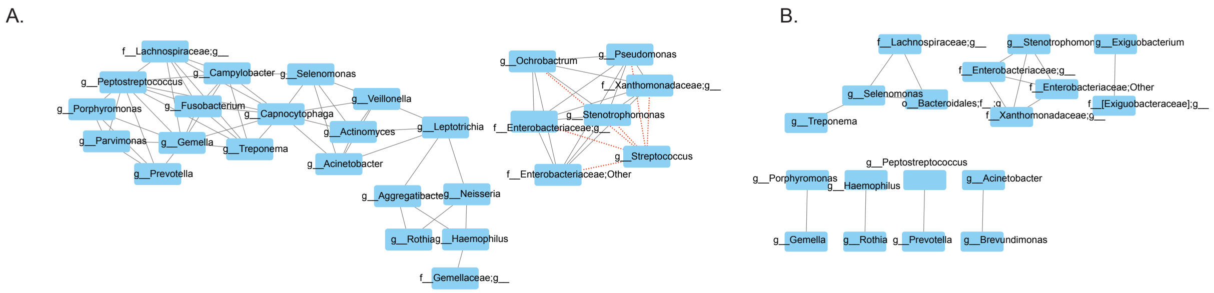

Supplemental figure 4: Co-occurrence analysis. Co-occurrence analysis of top 30 bacterial genera in (A) pre-cancer and (B) cancer groups with Pearson's correlation analysis presented through network analysis. Black line: positive interaction; red dotted line: negative interaction.

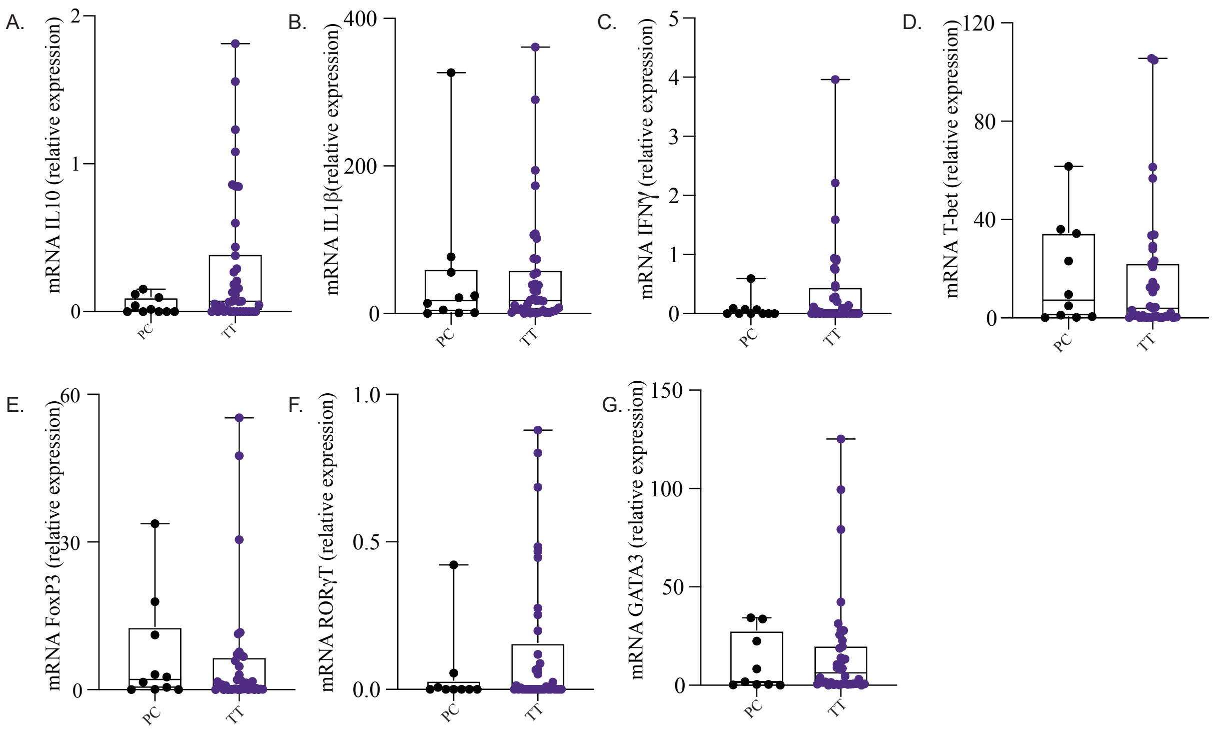

Supplemental figure 5: Relative expression of cytokine genes and T cell associated genes in pre-cancer and cancer group (A) *IL10* (B) *IL1β* (C) *IFNγ* (D) *T-bet* (T_H1) (E) *FoxP3* (Treg) (F) *RORγT* (T_H17) (G) *GATA3* (T_H2). n=10 (Pre-cancer group); n=40 (Cancer group). Statistical analysis was done t-test followed by Mann-Whitney test. PC: pre-cancer, TT-tumor tissue

Supplemental table 2: Dominant species having more than 2% of relative abundance.

Species	Relative abundances
Rothia__mucilaginosa	6.169
Capnocytophaga__granulosa	3.464
Capnocytophaga__leadbetteri	7.468
Capnocytophaga__ochracea	2.476
Capnocytophaga__sputigena	5.868
Exiguobacterium__aurantiacum	2.568
Streptococcus_dysgalactiae	2.992
Streptococcus__periodonticum	21.652
Fusobacterium__nucleatum	7.040
Brevundimonas__diminuta	5.058
Neisseria__mucosa	4.865
Neisseria__subflava	3.158
Haemophilus__parainfluenzae	5.236
Treponema__medium	3.525
Akkermansia__muciniphila	3.672

Supplemental table 3: Summary of the area under ROC curve (AUC) indices using differential genus in precancer and cancer samples.

Predictive Genus	AUC	95% CI	P value
Streptococcus	0.7874	0.6501 - 0.9246	0.0006
Rothia	0.7529	0.6267 - 0.8791	0.0027
Capnocytophaga	0.8103	0.6964 - 0.9243	0.0002
Treponema	0.7092	0.5758 - 0.8425	0.0130
Leptotrichia	0.6782	0.5260 - 0.8303	0.0343

95% CI;95% confidence interval

Supplemental table 4 -Significantly correlated TILs with most abundant genera of tumour microenvironment.

Tumour infiltrating lymphocytes	Genus	Pearson correlation coefficient (r)	P value
CD19	*Porphyromonas*	0.5847	0.0043
	Streptococcus	0.4902	0.0205
	Oscillospira	0.4607	0.031
	Aggregatibacter	0.4717	0.0267
CD 4	*Brevundimonas*	0.4939	0.0372
CD 4 Naive	*Rothia*	0.6261	0.0294
	Porphyromonas	0.8424	0.0006
	Prevotella	0.5869	0.0449
	Gemellaceae;g__	0.833	0.0008
	Streptococcus	0.6721	0.0167
	Oscillospira	0.6423	0.0243
	Aggregatibacter	0.6193	0.0318
CD 4 CM	*Porphyromonas*	0.7811	0.0027
	Prevotella	0.7172	0.0087
	Gemellaceae;g__	0.62	0.0315
	Oscillospira	0.5767	0.0496
CD 4 TEMRA	*Fusobacterium*	0.8277	0.0009
	Leptotrichia	0.7631	0.0039
	Capnocytophaga	0.7192	0.0084
	c__BD1-5;o	0.9427	<0.0001
CD 4 EM	*Porphyromonas*	-0.6775	0.0155
	Prevotella	-0.647	0.023

	Exiguobacterium	0.6118	0.0345
	Gemellaceae;g__	-0.6679	0.0176
	Fusobacterium	-0.6682	0.0175
	Campylobacter	-0.8084	0.0015
CD 8 Naïve	*Porphyromonas*	0.7569	0.0044
	F_Gemellaceae;g__	0.8362	0.0007
	Gemella	0.6605	0.0194
	Streptococcus	0.8303	0.0008
CD 8CM	*Campylobacter*	0.5851	0.0457
CD 8 TEMRA	*Pedobacter*	0.6351	0.0265
	Leptotrichia	-0.58	0.0481
	Capnocytophaga	-0.5694	0.0533
CD 8 EM	*Porphyromonas*	-0.6252	0.0297
	F_Gemellaceae;g__	-0.6108	0.0349
	Gemella	-0.6287	0.0285
	Streptococcus	-0.7388	0.0061

CM; Central memory: EM; Effector memory: TEMRA: Effector memory cell re-expressing CD45RA

Tumour-infiltrating lymphocytes percentage value of flow cytometry data were correlated with relative abundances of intra-tumoral bacteria at genus level with Pearson's correlation parameter. Values with Pearson's co-relation coefficient (r) >0.5 and p value <0.05 are shown in the table.

Supplemental Table 5- List of antibodies used in flowcytometry and IHC.

Antibody	Clone	Sources
CD 19-FITC	HIB19	BD Biosciences
CD 3-PERCP	SK7	BD Biosciences
CD 4-FITC	RPA-T4	BD Biosciences
CD 8-PE	RPA-T8	BD Biosciences
CD 45RA-APC	HI100	BD Biosciences
CD 45RO-APC H7	UCHL1	eBioscience
CCR7- PE-Cyanine7	3D12	eBioscience
CD24-PE	ML5	BD Biosciences
CD27-PECY7	M-T271	BD Biosciences
CD38-APC	HIT2	BD Biosciences
CD20cy	L26	Dako
CD3	-	Dako
IgA	-	Dako

Supplemental Table 6 - Primers used for RT- qPCR.

Gene name	Primer sequence
IL-1β	CGCCAATGACTCAGAGGAAG
	AGGGCGTCATTCAGGATCAA
IL 6	GTAGCCGCCCCACACAGACAGCC
	GCCATCTTTGGAAGGTTC
IFNγ	TCAGCTCTGCATCGTTTTGG
	GTTCCATTATCCGCTACATCTGAA
TNFα	TCTTCTCGAACCCCGAGTGA
	CCTCTGATGGCACCACCAG
IL 10	GTGATGCCCCAAGCTGAGA
	CACGGCCTTGCTCTTGTTTT
TGF-β	CAGCAACAATTCCTGGCGATA
	AAGGCGAAAGCCCTCAATTT
T-bet	CCCCTTGGTGTGGACTGAGA
	ACGCGCCTCCTCTTAGAGTC
GATA-3	GTCCTCCCTGAGCCACATCT
	GTGGTCCAAAGGACAGGCTG
RORγ t	CGCTCCAACATCTTCTCC
	CTAACCAGCACCACTTCC
Foxp3	CAGCCATGATCAGCCTCACA
	GCACTGGGATTTGGGAAGGT
GAPDH	CCTGCACCACCAACTGCTTA
	GGCCATCCACAGTCTTCTGAG
